# LINC00460 Hypomethylation Promotes Metastasis in Colorectal Carcinoma

**DOI:** 10.3389/fgene.2019.00880

**Published:** 2019-09-30

**Authors:** Hui Zhang, Ya Lu, Jianzhong Wu, Jifeng Feng

**Affiliations:** Research Center for Clinical Oncology, The Affiliated Cancer Hospital of Nanjing Medical University, Jiangsu Cancer Hospital, Jiangsu Institute of Cancer Research, Nanjing, China

**Keywords:** DNA methylation, LINC00460, CRC, metastasis, CpG island

## Abstract

**Introduction:** Epigenetic alterations and aberrantly expressed long noncoding RNAs (lncRNAs) are pervasive in colorectal cancer (CRC) tumorigenesis. DNA methylation could control lncRNA expression and play an important role in tumor initiation and progression. However, the DNA methylation that regulates lncRNAs in CRC remains poorly characterized.

**Materials and Methods:** In our research, we integrated dysregulated expression and methylation of lncRNAs between colorectal tumor and adjacent mucosa tissues from The Cancer Genome Atlas database. With the use of this strategy, LINC00460, the most frequently epigenetically activated, was identified and further verified in the Cancer Cell Line Encyclopedia and Gene Expression Omnibus databases.

**Results:** Patients with high expression of LINC00460 are prone to metastasis and are associated with poor prognosis. Abnormally expressed LINC00460 could be used as an independent prognostic risk factor for disease-free survival. Knockdown of LINC00460 promotes colon cancer cell invasion and migration *in vitro*.

**Conclusion:** In summary, our results suggest that DNA methylation-regulated LINC00460 could promote CRC metastasis and serve as a potential therapeutic target for CRC.

## Introduction

As one of the most common malignancies, colorectal cancer (CRC) ranks third (11.6% of total cases) for incidence and second (9.2%) for mortality worldwide (Bray et al., 2018). The global burden of CRC is expected to increase by 60% to more than 2.2 million new cases and 1.1 million cancer deaths by 2030 (Arnold et al., 2017). Lymph node and distant metastases are frequently observed in patients, leading to frustrating treatment (Sasaki et al., 2008). The combination of chemotherapy regimens and targeted therapies has prominently improved the survival rate for patients with metastasis; however, the 5-year survival rate remains below 15% (Brenner et al., 2014). Therefore, developing novel biomarkers and treatment strategies are urgently needed. The aberrant expression of long noncoding RNAs (lncRNAs) plays critical roles in multiple biological processes of CRC, such as tumorigenesis, proliferation, and metastasis (Ragusa et al., 2015; Weng et al., 2016). Numerous lncRNAs such as H19 (Ohtsuka et al., 2016), CCAT1 (McCleland et al., 2016), HOTAIR (Kogo et al., 2011), MALAT-1 (Ji et al., 2014), DACOR1 (Shi et al., 2015), and PVT-1 (Takahashi et al., 2014) are dysregulated in CRC and may serve as therapeutic targets for CRC. Similar to protein-coding genes, lncRNA expression is subject to changes, such as copy-number alterations (Hu et al., 2014), cancer risk polymorphism (Guo et al., 2016), and epigenetic regulation (Wang Z et al., 2018), which occur in tumorigenesis. CRCs are characterized by global DNA hypomethylation and promoter-specific DNA methylation associated with genomic instability and tumor initiation (Tse et al., 2017). However, the DNA methylation of lncRNA genes in CRC remains poorly characterized. As the most authoritative cancer genomic/epigenetic projects, The Cancer Genome Atlas (TCGA) database (http://tcga-data.nci.nih.gov/tcga/) provides us valuable opportunity to characterize the lncRNA epigenetic landscape in CRC. In our study, we integrated the lncRNA RNA-seq and DNA methylation data of CRC in the TCGA database. The aberrantly expressed lncRNAs targeted by methylation in CRC may play an important role in tumor occurrence and progression. The most frequently methylation regulated was LINC00460, which was identified and verified in the Cancer Cell Line Encyclopedia (CCLE) (https://portals.broadinstitute.org/ccle) and Gene Expression Omnibus (GEO) databases (https://www.ncbi.nlm.nih.gov/pubmed). Knockdown of LINC00460 promote colon cancer cell invasion and migration *in vitro*. In summary, our results suggest that the hypomethylated oncogenic LINC00460 could promote CRC metastasis and serve as a potential therapeutic target for CRC.

## Materials and Methods

### Data Collection and Bioinformatics Analysis

CRC RNA-seq and DNA methylation data were downloaded from the TCGA database. In this study, 407 CRC tumors and 21 adjacent mucosa tissues with RNA-seq and HM450 DNA methylation data were enrolled. The abnormally expressed lncRNAs (|logFC| > 5, *P* < 0.01) between tumor and adjacent normal tissue were screened out. Pearson correlation coefficients between methylation alteration and gene expression for each lncRNA were calculated. LncRNA with coefficient >0.3 and *P* < 0.05 were considered significant and selected as candidate molecule. To further clarify the correlation between methylation and gene expression, we downloaded RNA-seq data and HM450 DNA methylation data of LINC00460 in 706 cancer cell lines (17 kinds of tumor) in CCLE database. Furthermore, three colon cancer cells (HCT116, LOVO, and SW620) were treated with 5‐aza‐2′‐deoxycytidine (Sigma‐Aldrich, St. Louis, MO) for 48 h. LINC00460 expression was analyzed by quantitative real-time PCR (qPCR).

### CpG Island Methylator Phenotype Status of LINC00460

The CpG island DNA methylation level located at 3 kb upstream of the lncRNA promoter region was extracted from the TCGA database. Using a similar strategy, the CpG islands significantly related to lncRNA expression were filtered out and further verified in the GEO database.

### LINC00460 Survival Prediction Ability and Its Relationship With Clinicopathological Characteristics

We inferred that the methylated lncRNA may play an important role in CRC tumorigenesis. Hence, we subsequently examined the expression of LINC00460 in normal, tumor, and metastatic tissues in GEO database. Furthermore, the relationship between LINC00460 expression and clinical pathology data (age, gender, American Joint Committee on Cancer TNM stage, T stage, lymph node status, and metastasis) in GSE14333 was evaluated. Kaplan–Meier survival analysis with log-rank test was performed to determine the cumulative overall survival (OS) and disease-free survival (DFS) rate of patients with CRC. Then, patients were divided into two groups according to the median of LINC00460 expression. Uni- and multivariate analyses were used to assess the ability of LINC00460 expression as an independent prognostic risk factor.

### Pathway Enrichment Analysis

To get further insights into the mechanism of LINC00460 in CRC tumorigenesis, we performed Kyoto Encyclopedia of Genes and Genomes (KEGG) pathway enrichment analysis for the target genes of linc00460 in the Database for Annotation, Visualization, and Integrated Discovery (Huang et al., 2009a; Huang et al., 2009b). These target genes were predicted from Multi Experiment Matrix (MEM) database (Adler et al., 2009; Kolde et al., 2012) (https://biit.cs.ut.ee/mem/), and the analysis results were visualized by Cytoscape 3.6.0 (Su et al., 2014). *P* < 0.05 was regarded as significant.

### Cell Culture, siRNA, and Transfection

Human CRC cell lines HCT116, SW620, SW480, and LOVO and normal colonic epithelial cell NCM460 were purchased form the American Type Culture Collection (ATCC, Manassas, Virginia, USA) and cultured in Dulbecco’s modified Eagle’s medium (KeyGEN BioTECH, Jiangsu, China) with 10% fetal bovine serum (Gibco, USA). The cells were incubated at 37°C in a 5% CO_2_ incubator. LINC00460 Smart Silencer was synthesized by Guangzhou RiboBio (Guangzhou, China). Transfection was performed using Lipofectamine RNAiMAX (Invitrogen, USA) following the instructions of the manufacturer.

### RNA Isolation, Reverse Transcription, and qPCR

Total RNA from cells was extracted by TRIzol reagent (Invitrogen, USA) and reverse transcribed using PrimeScript RT Master Mix (Takara, Japan) according to the instruction manual. qPCR was performed using ABI 7300 Real‐Time PCR System (ABI, CA) and SYBR Green Master Mix (Thermo Fisher Scientific, CA). lncRNA expression was normalized to glyceraldehyde 3-phosphate dehydrogenase (GAPDH) (ΔCt = target lncRNA Ct − GAPDH Ct). Primer used were GAPDH, 5′-GGTGAAGGTCGGAGTCAACG-3′ and 5′- TGGGTGGAATCATATTGGAACA-3′; LINC00460, 5′- GTGGATGAGAACGAAGGTTACG-3′ and 5′-CTTTCCCACGCTCAGTCTTT-3′.

### Migration and Invasion Assays

Cell migration and invasion assays were performed using Transwell chambers (8-mm pores, Corning, USA) precoated without (migration assay) or with (invasion assay) Matrigel (BD Biosciences). First, the upper chambers were plated with 5 × 10^4^ cells in 200 µl of serum-free medium, whereas the lower chambers were filled with 500 µl of medium supplemented with 10% fetal bovine serum. After 36 h of incubation, the cells on the upper surface of the membrane filter were fixed with methyl alcohol and stained with hematoxylin. The number of cells that had migrated or invaded was counted and imaged under an inverted microscope.

### Wound Healing Cell Migration Assays

HCT116 and SW480 were seeded into six-well plates at a density of 5 × 10 5 cells per well. After 24 h of incubation, scratched wounds were made using sterile 10-μl pipette tips through a premarked line. The specific wound areas, over or under premarked lines, were displayed at 0 and 48 h by taking images under the optical microscope.

### CCK8 Assay

For the CCK8 cell proliferation assay, 1 × 10^4^ cells were seeded onto 96-well plates and cultured for 4 days. Cell viability was assessed daily using CCK8 staining method according to the manufacturer’s instructions (Dojindo, Japan). Absorbance was measured at 450 nm using a byspectrophotometric plate reader (BioTek ELX800, USA).

### Statistical Analysis

The differential lncRNA screening was performed using “edgeR” package and drawn with “ggplot2” package in R 3.5.1. The DNA methylation and lncRNA expression between tumor and adjacent normal tissues were analyzed using *t* test. Standard ANOVA was completed for the three groups. The correlation between LINC00460 expression and clinicopathological data was obtained using chi-square test. LncRNA expression from TCGA was transformed by log2. The following R packages were used in our study: “pheatmap,” “hash,” “limma,” “survival,” and “corrplot.”

## Results

### Epigenetic Landscape of lncRNAs in CRC Revealed LINC00460 as a Candidate Gene

**Figure 1:**
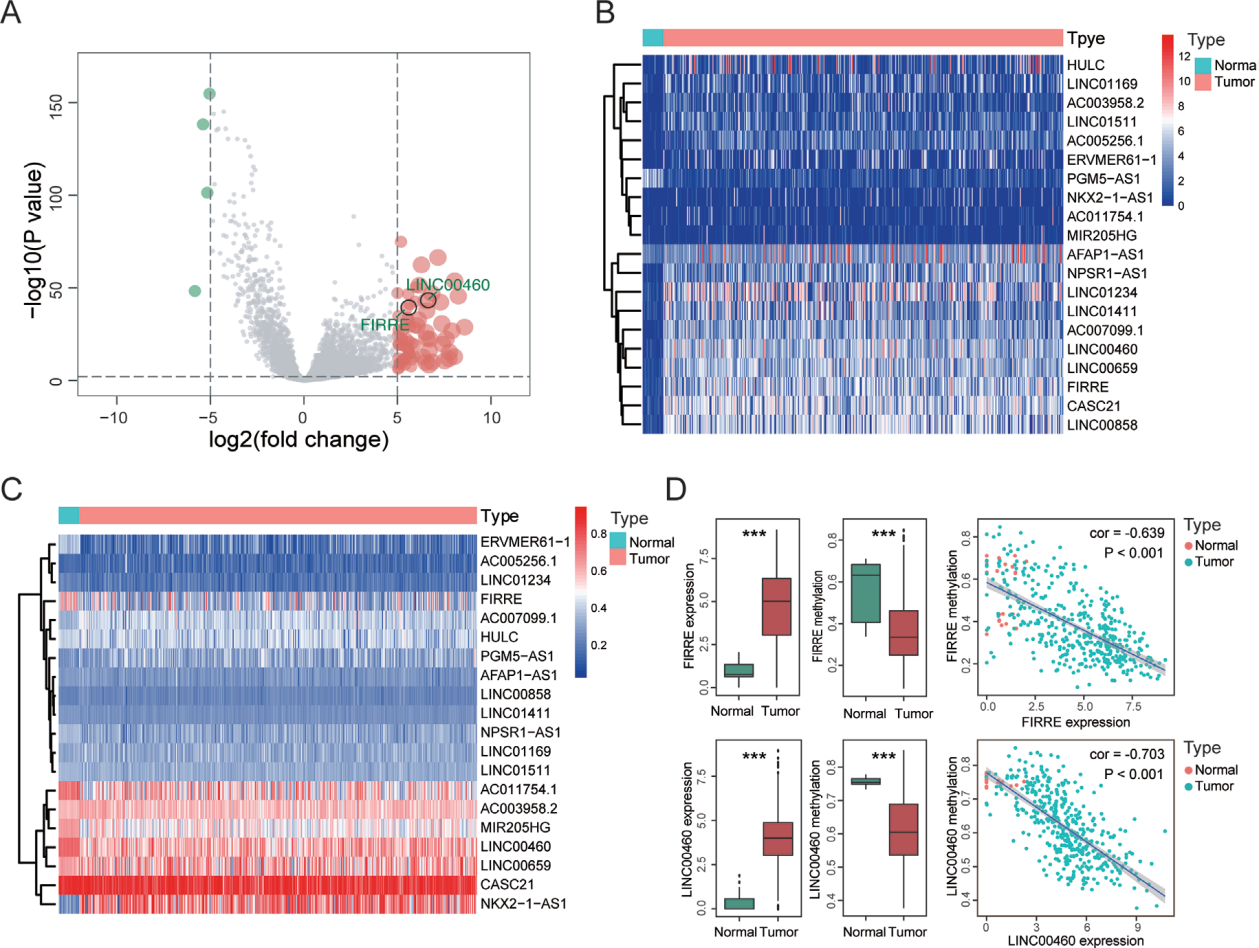
Epigenetic landscape of long noncoding RNAs (lncRNAs) in colorectal cancer (CRC) revealed LINC00460 as a candidate gene. **(A)** Volcano of 75 different expressed lncRNAs between tumor and adjacent normal tissues from The Cancer Genome Atlas (TCGA) database. Heatmap of 20 lncRNAs which contained both expression **(B)** and methylation **(C)** data. **(D)** Pearson correlation between expression and methylation of two hypomethylated lncRNAs (LINC00460 and FIRRE). ***p < 0.001.

To identify the differential expressed lncRNAs between CRC and normal tissues, we analyzed the TCGA database and screened 75 differently expressed (71 upregulated and 4 downregulated) lncRNAs according to the criteria (**Figure 1A** and **Supplementary Table 1**). We selected 20 lncRNAs containing methylation and expression information (**Figures 1B, C** and **Table 1**). By performing Pearson correlation analysis, we obtained two hypomethylated lncRNAs (FIRRE and LINC00460) (correlation coefficient = −0.639 and −0.703, respectively, **Figure 1D**). Furthermore, these two lncRNAs had different expression and methylation levels in tumor and adjacent normal tissues (**Figure 1D**), indicating their consistent role in the tumor process. We selected LINC00460 for further research due to its remarkably alternation in CRC.

**Table 1:** Twenty long noncoding RNAs (lncRNAs) containing both methylation and expression screened from The Cancer Genome Atlas (TCGA) database.

LncRNAs	Expression			Methylation		
	Normal	Tumor	*P* value^a^	Normal	Tumor	*P* value^b^
AC003958.2	0.036 ± 0.165	1.562 ± 1.818	<0.001	0.591 ± 0.117	0.583 ± 0.612	0.564
AC005256.1	0.131 ± 0.415	2.270 ± 1.742	<0.001	0.158 ± 0.146	0.163 ± 0.536	0.618
AC007099.1	0.118 ± 0.297	3.950 ± 1.970	<0.001	0.345 ± 0.029	0.394 ± 0.074	0.003
AC011754.1	0.177 ± 0.656	0.560 ± 1.234	0.158	0.733 ± 0.404	0.540 ± 0.154	<0.001
AFAP1-AS1	2.505 ± 0.928	4.508 ± 2.827	0.001	0.281 ± 0.118	0.269 ± 0.405	0.171
CASC21	0.809 ± 0.929	5.249 ± 2.010	<0.001	0.875 ± 0.024	0.848 ± 0.067	0.063
ERVMER61-1	0.045 ± 0.206	1.509 ± 2.097	0.004	0.370 ± 0.037	0.198 ± 0.095	<0.001
FIRRE	0.855 ± 0.581	4.681 ± 2.193	<0.001	0.554 ± 0.141	0.373 ± 0.158	<0.001
HULC	0.353 ± 0.767	2.493 ± 3.240	0.003	0.414 ± 0.019	0.387 ± 0.086	0.161
LINC00460	0.376 ± 0.687	4.806 ± 1.907	<0.001	0.756 ± 0.013	0.615 ± 0.099	<0.001
LINC00659	0.729 ± 0.784	4.587 ± 1.893	<0.001	0.633 ± 0.032	0.608 ± 0.148	0.439
LINC00858	0.662 ± 0.809	4.832 ± 2.388	<0.001	0.226 ± 0.008	0.234 ± 0.027	0.203
LINC01169	0.269 ± 0.511	2.045 ± 2.184	<0.001	0.316 ± 0.237	0.320 ± 0.052	0.767
LINC01234	0.788 ± 0.768	4.766 ± 3.167	<0.001	0.224 ± 0.172	0.188 ± 0.471	0.001
LINC01411	0.634 ± 0.807	3.788 ± 2.672	<0.001	0.280 ± 0.066	0.260 ± 0.018	<0.001
LINC01511	0.199 ± 0.458	1.951 ± 1.828	<0.001	0.350 ± 0.069	0.325 ± 0.034	0.001
MIR205HG	0.121 ± 0.404	0.424 ± 1.053	0.190	0.647 ± 0.158	0.494 ± 0.089	<0.001
NKX2-1-AS1	0.463 ± 0.212	0.637 ± 1.504	0.073	0.256 ± 0.622	0.680 ± 0.167	0.002
NPSR1-AS1	0.307 ± 0.537	3.549 ± 2.252	<0.001	0.358 ± 0.113	0.307 ± 0.629	<0.001
PGM5-AS1	4.578 ± 1.723	1.027 ± 1.149	<0.001	0.260 ± 0.414	0.320 ± 0.081	<0.001

a Difference of lncRNAs expression between tumor and normal.

b Difference of lncRNAs methylation between tumor and normal.

### LINC00460 Overexpressed in CRC Is Activated by DNA Methylation

**Figure 2:**
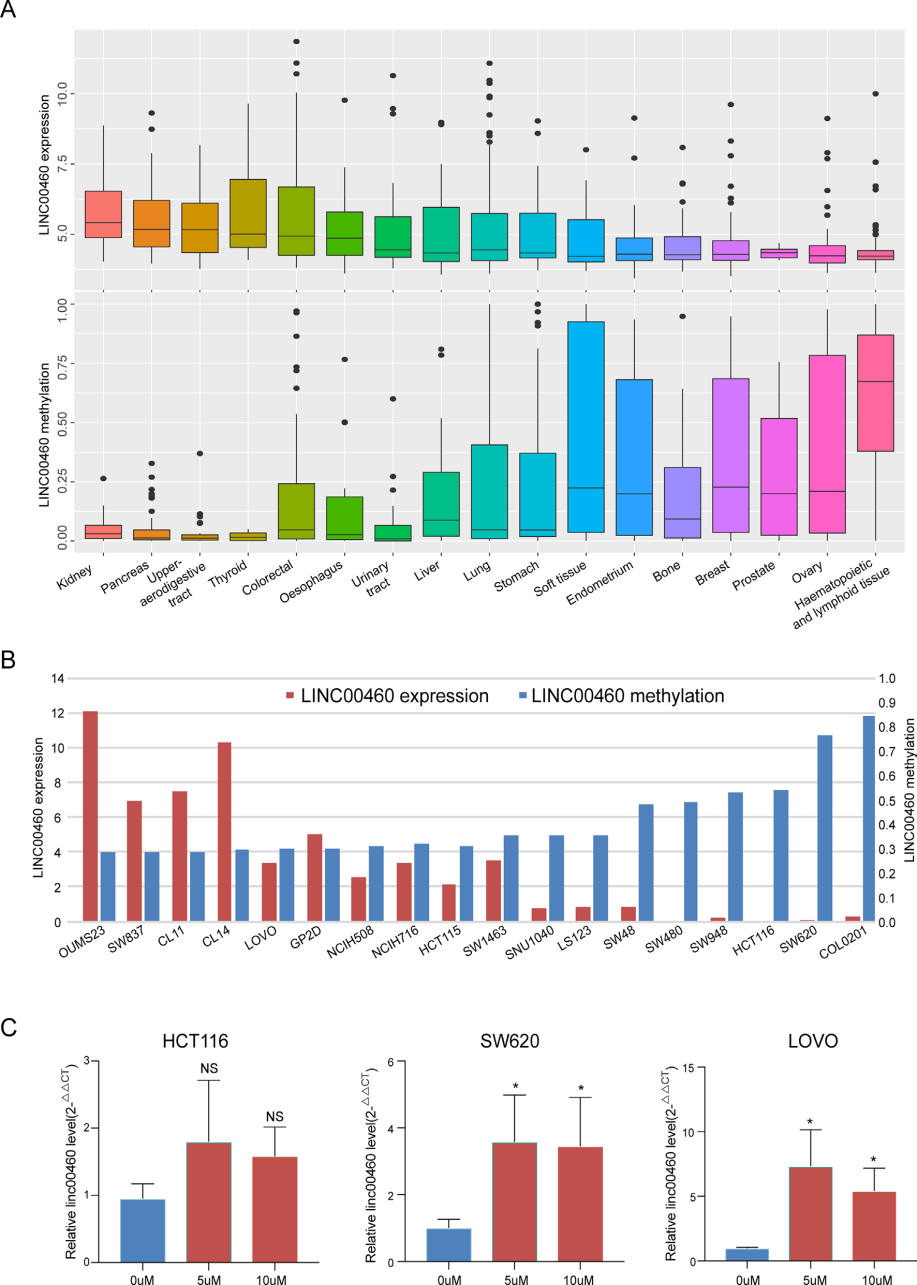
LINC00460 overexpressed in CRC was activated by DNA methylation. LINC00460 expression and DNA methylation status in 17 kinds of tumor cells **(A)** and 18 CRC cell lines **(B)**. **(C)** Quantitative real-time PCR (qPCR) analysis of LINC00460 expression in HCT116, LOVO, and SW620 treated with 5-aza-2′-deoxycytidine. *p < 0.05.

By investigating the RNA-seq and HM450 DNA methylation profiles of 17 kinds of tumor cell lines from the CCLE database, we obtained a significant negative correlation between LINC00460 expression levels and its promoter methylation (**Figure 2A**). Furthermore, similar correlations were observed in 18 CRC cell lines (**Figure 2B**). Three colon cancer cells (SW620, HCT116, and LOVO) were treated with 5‐aza‐2′‐deoxycytidine for 48 h, and the LINC00460 expression was observed. Although no significant change was observed in HCT116, 5-aza treatment caused LINC00460 overexpression and demethylation in LOVO and SW620. Therefore, 5 µM was an effective concentration in our experiments.

### CpG Island Methylator Phenotype Status of LINC00460

**Figure 3:**
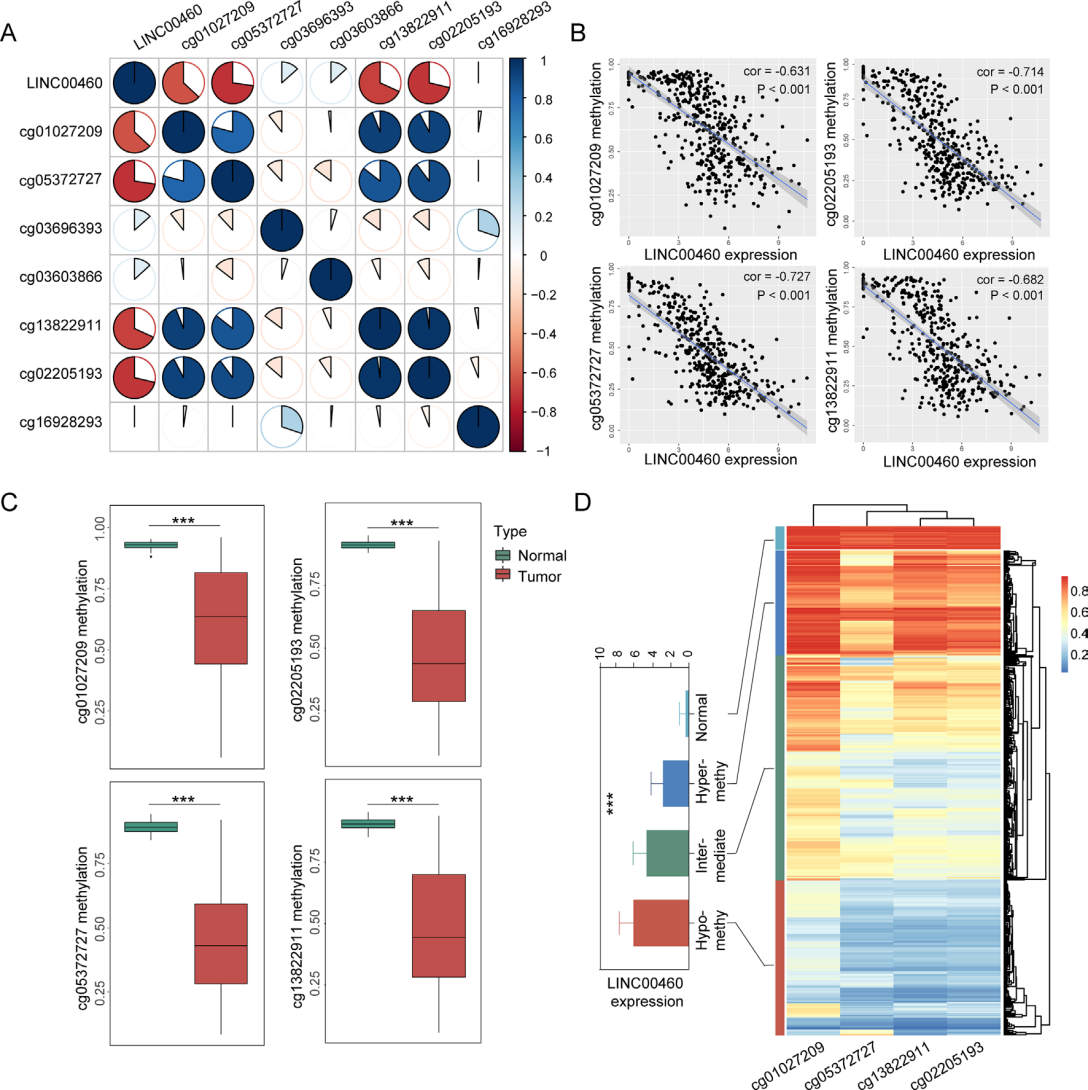
CpG island methylation of LINC00460. **(A)** Correlation between seven CpG methylation islands and LINC00460 expression screened from TCGA database. **(B)** Four islands showing a significant negative correlation with LINC00460 expression. **(C)** CpG island methylation statue between tumor and normal. **(D)** Heatmap of four CpG sites methylation in LINC00460 promoter. Three subgroups were identified by hierarchical clustering analysis in tumor. The DNA methylation in normal tissue is shown as the control. ***p < 0.001.

**Figure 4:**
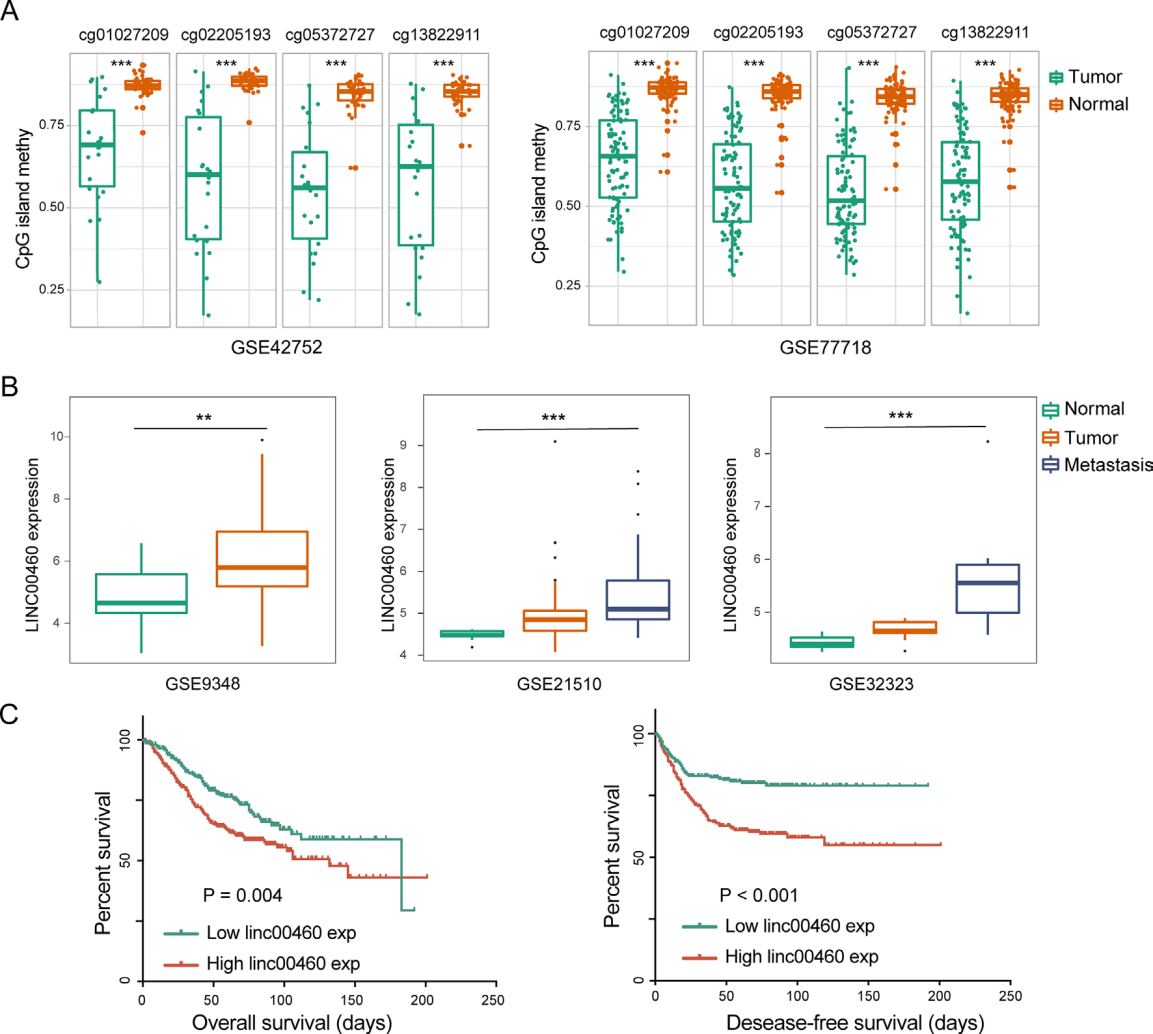
LINC00460 was correlated with metastasis and poor survival in CRC. **(A)** Abnormal methylation of four CpG islands between tumor and normal tissue in GSE42752 and GSE77718. **(B)** LINC00460 expression in GSE9348, GSE21510, and GSE32323. CRC patients with distant metastasis and a high expression of LINC00460. **(C)** Association between LINC00460 expression and CRC survival [overall survival (OS) and disease-free survival (DFS)] in GSE14333. **p < 0.01, ***p < 0.001.

From the seven CpG sites of methylation analysis located at the upstream of the transcription start site of the LINC00460 from TCGA database, four showed a significant negative correlation with LINC00460 expression (**Figures 3A, B**). In addition, the methylation levels of these CpGs were significantly different between tumor and normal tissues (**Figure 3C** and **Table 2**). According to the selected CPG islands, a heatmap was drawn, and three subgroups were identified by hierarchical clustering analysis in tumor. Compared with that in the normal group, the LINC00460 in tumor tissue was significantly hypomethylated, which was negatively correlated with its expression (**Figure 3D**). The abnormal methylation of these four CpG sites were further verified in GSE42752 and GSE77718 (**Figure 4A**).

**Table 2:** Pearson correlation between CpG islands methylation and LINC00460 expression.

CpG islands	Methylation			LINC00460 correlation	
	Normal	Tumor	*P* value^a^	COR	*P* value^b^
cg01027209	0.928	0.623	<0.001	−0.631	<0.001
cg05372727	0.893	0.451	<0.001	−0.727	<0.001
cg03696393	0.979	0.983	0.144	0.134	0.005
cg03603866	0.8638	0.872	0.358	0.132	0.006
cg13822911	0.905	0.487	<0.001	−0.682	<0.001
cg02205193	0.911	0.476	<0.001	−0.714	<0.001
cg16928293	0.981	0.984	0.403	0.002	0.961

COR, correlation coefficients.

a ^a^Difference of CpG islands methylation between tumor and normal.

b Pearson correlation between CpG islands methylation and LINC00460 expression.

### Dysregulated LINC00460 Promoted CRC Metastasis and Was Correlated With Poor Survival in CRC

**Figure 5:**
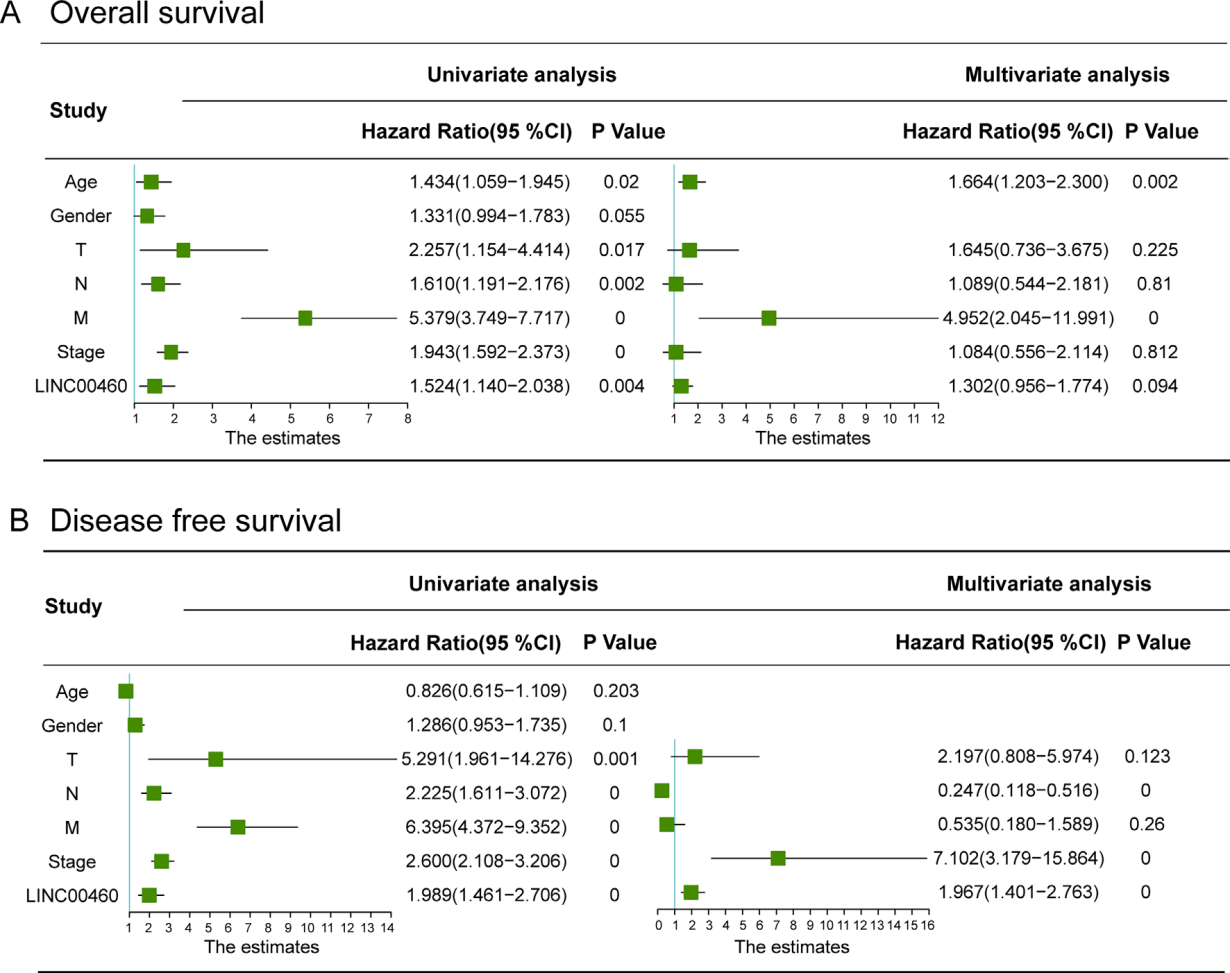
Univariate and multivariate Cox regression analyses of overall survival **(A)** and disease free survival **(B)** in CRC patients. LINC00460 expression could be used as an independent prognostic risk factor for CRC disease-free survival.

As shown in **Figure 4B**, LINC00460 was dysregulated in GSE9348, GSE21510, and GSE32323. Furthermore, patients with CRC and distant metastasis had a high expression of LINC00460. Overexpression of LINC00460 was positively associated with advanced AJCC TNM stage (*P* = 0.013), T stage (*P* = 0.023), and lymph node status (*P* = 0.006) (**Table 3**) in GSE14333. We further determined whether or not the aberrant expression of LINC00460 could affect the prognosis of patients. Patients with high expression of LINC00460 have poor OS and DFS (*P* < 0.05) (**Figure 4C**). In the univariate cox’s proportional hazards models, LINC00460 could affect the OS and DFS of patients with CRC. Multivariate analysis revealed that LINC00460 expression could be used as an independent prognostic risk factor for DFS (**Figure 5**).

**Table 3:** Correlations between LINC00460 expression and clinicopathological characteristics.

Variable	Number	LINC00460 expression		
		Low exp	High exp	*P* value
**Age**				
≤65	227	114	113	0.905
> 65	346	172	174	
**Gender**				
Female	257	127	130	0.802
Male	317	160	157	
**T stage**				
T1 + T2	63	40	23	0.020
T3 + T4	491	237	254	
**Lymph node status**				
N0	309	171	138	0.006
N1–2	239	104	135	
**Metastasis**				
M0	491	248	243	0.498
M1	61	28	33	
**Stage**				
I	41	24	17	0.013
II	267	147	120	
III	206	89	117	
IV	60	27	33	

### Pathway Enrichment Analysis

**Figure 6:**
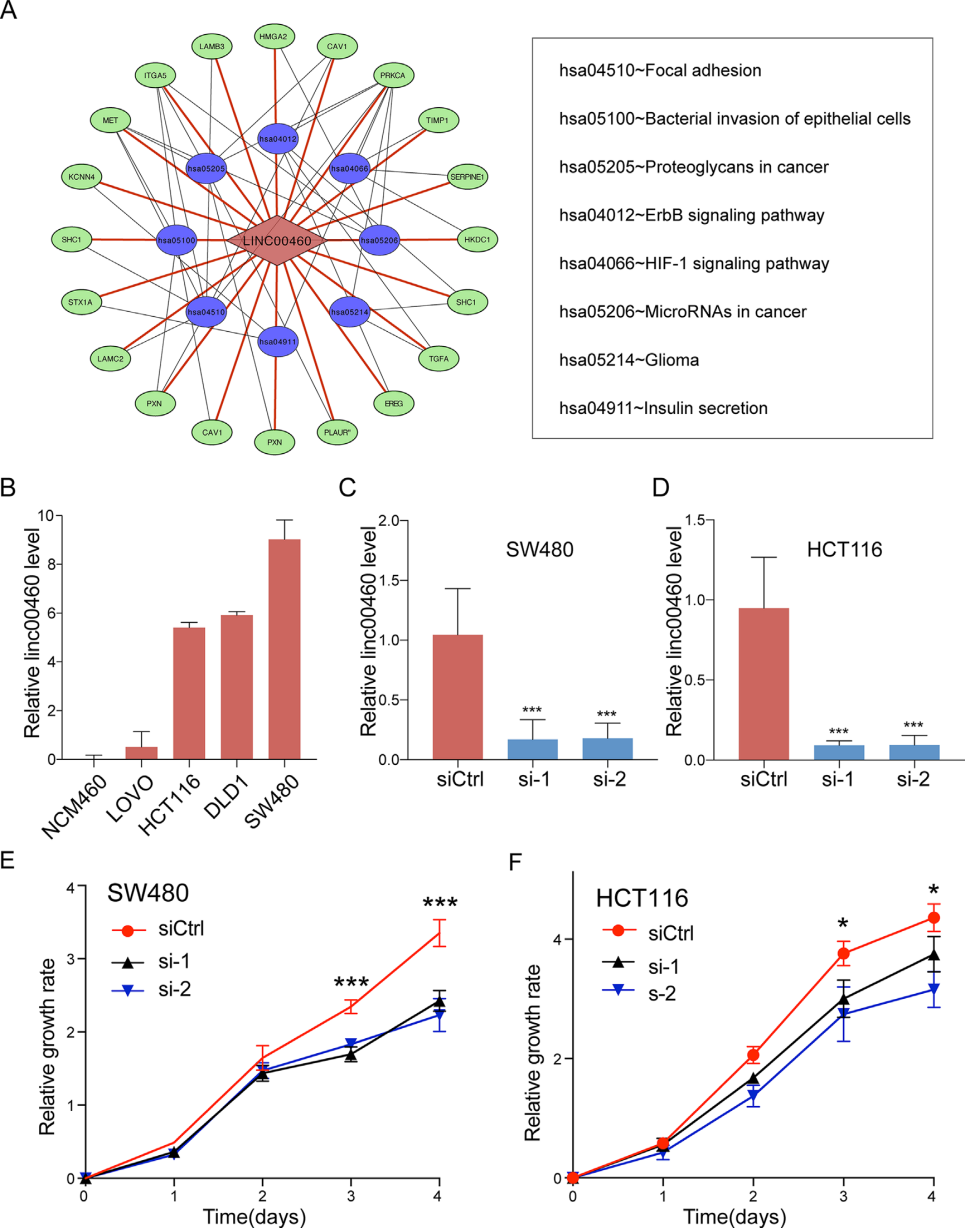
Pathway enrichment analysis. **(A)** Statistically significant KEGG pathway for the target genes of LINC00460. **(B)** LINC00460 expression in human CRC cell lines. SW480 **(C)** and HCT116 **(D)** were transfected with LINC00460 siRNA1 and siRNA2 or scramble control siRNA for 48 h, and the expression level was detected by qPCR. Cell viability was determined by CCK-8 assay in SW480 **(E)** and HCT116 **(F)**. SiCtrl, Control group; si-1, si-2, LINC00460 Smart Silencer-1 and -2. *p < 0.05, ***p < 0.001.

To get further insights into the mechanism of LINC00460 in CRC tumorigenesis, we performed KEGG pathway enrichment analysis for the target genes of LINC00460 as predicted by MEM database (**Supplementary Table 2**). The results were visualized by Cytoscape and are shown in **Figure 6A**. *P* < 0.05 was regarded as significant.

### LINC00460 Promoted CRC Cell Metastasis *in Vitro*

**Figure 7:**
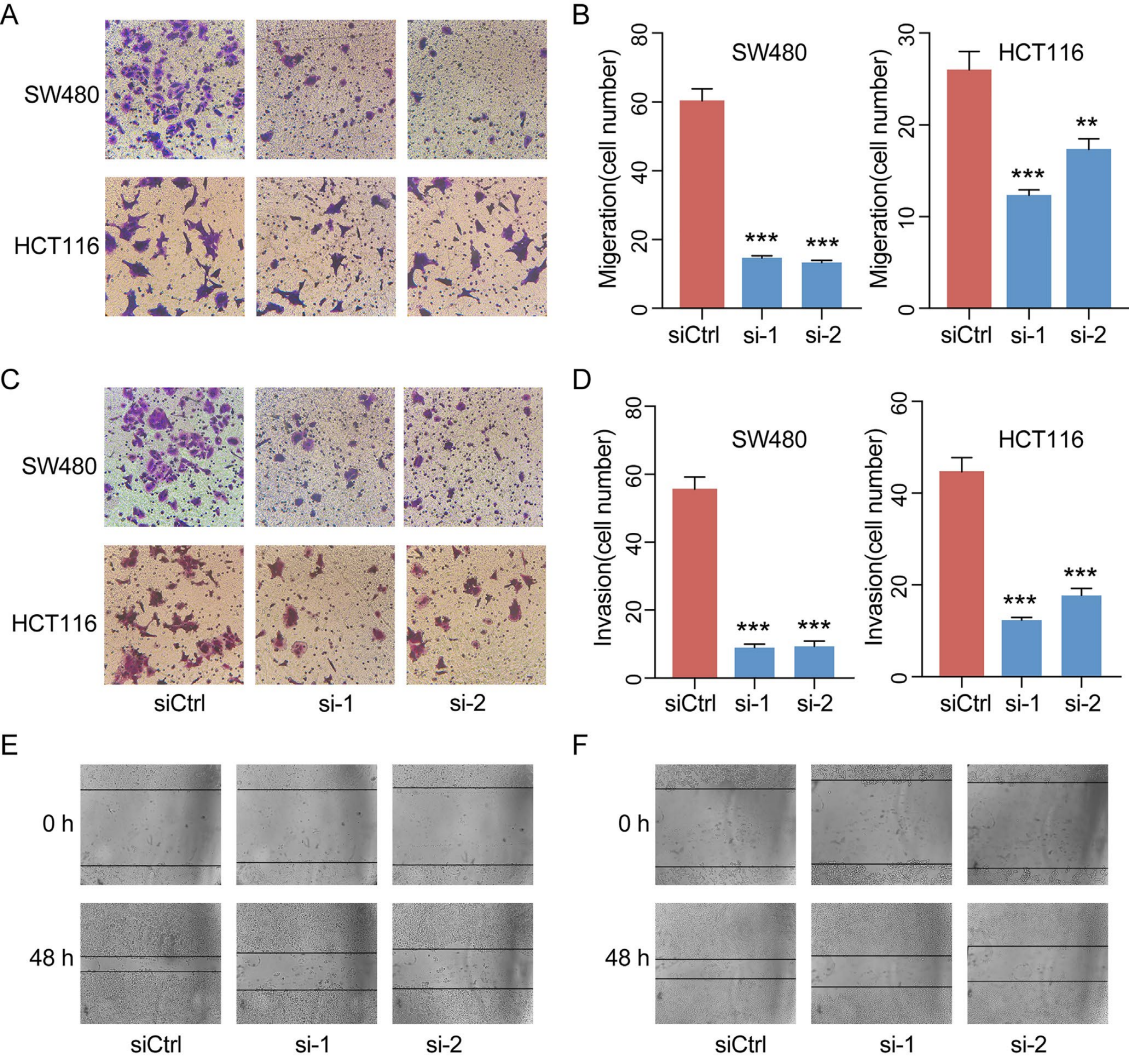
LINC00460 promoted CRC cell migration and invasion. Representative images of the migrated cells **(A)** and invaded cells **(C)** between control and siLINC00460. **(B, D)** Quantifications of migrated and invaded cells in Figures C and E. **P < 0.01, ***P < 0.001. Cell migration was further analyzed by scratch assay. SW480 **(E)** and HCT116 **(F)** cells were seeded in six-well plates and wounded by a 10-μl pipette tip. Then, the cells were grown for 48 h for photo recording. **p < 0.01, ***p < 0.001.

Given the significant correlations between the increased LINC00460 expression and CRC in multiple GEO datasets, we hypothesized that LINC00460 might promote CRC cell proliferation and metastasis. QRT-PCR results revealed that the expression of LINC00460 is overexpressed in diverse CRC cells including SW480, HCT116, SW620 and LOVO as compared with NCM460 (**Figures 6B**). Ectopic expression of LINC00460 could relatively promote cell growth as evidenced by CCK8 assays in HCT116 and SW480 (**Figures 6C-F**). However, Transwell assays with or without Matrigel showed that LINC00460 knockdown remarkably repressed SW480 and HCT116 cell migration (**Figures 7A, B**) and invasion (**Figures 7C, D**). The migration ability of LINC00460 was further proved by wound healing test (**Figures 7E, F**). In summary, our results revealed that LINC00460 knockdown could inhibit CRC metastasis *in vitro*.

## Discussion

As one of the most common and deadly cancers in the world, CRC incidence and mortality have increased in China (Chen et al., 2016); therefore, identifying novel diagnostic biomarker and therapeutic has become imperative. Numerous studies have suggested that alterations of lncRNA play vital roles in tumorigenesis (Prensner and Chinnaiyan, 2011; Schmitt and Chang, 2016), suggesting their potential as a novel target for CRC diagnosis and therapy. Epigenetic alterations play a role in initiation and progression and could be used as hallmark of tumors (Shen and Laird, 2013; Ren et al., 2017). Dysregulated global methylation and CpG island methylator phenotype are extensively observed in CRC (Tse et al., 2017). The expression of lncRNA could be regulated by epigenetics (Amin et al., 2015; Wang Z et al., 2018). However, the aberrant methylation events of lncRNAs and their consequences in CRC must be investigated further.

In our study, we identified differentially expressed lncRNAs between CRC and normal tissues from TCGA. Pearson correlation coefficients were calculated for the methylation alteration and gene expression for each lncRNA. Using this strategy, we successfully identified LINC00460 as the most frequently epigenetically activated. Further promoter-specific methylation analysis helped us identified four probes that mapped to the LINC00460 CpG islands. We validated the global and CpG island methylation of LINC00460 in the GEO and CCLE databases to further reveal its abnormal regulation and their potential role in tumor formation. We inferred that the aberrantly methylated lncRNA may play an important role in CRC occurrence and progression.

The overexpression of LINC00460 could promote cell proliferation and invasion in lung cancer (Li et al., 2018; Ye et al., 2019), gastric cancer (Wang F et al., 2018), ovarian cancer (Liu X et al., 2018), esophageal cancer (Liu W et al., 2018), and gefitinib resistance in nonsmall cell lung cancers (Ma et al., 2019), thus suggesting its carcinogenic effect. However, its role in colon cancer remains unclear (Lian et al., 2018; Wang X et al., 2018; Zhang et al., 2019). Wang’s study indicated that the abnormal expression of LINC00460 could inhibit proliferation but had no effect on migration or invasion. However, Lian’s research indicates that LINC00460 could promote cell proliferation. Consistent with Zhang’s findings, our results indicated that the knockdown of LINC00460 could inhibit CRC proliferation, suggesting its carcinogenesis effect on the tumorigenesis of CRC. Dysregulated LINC00460 significantly affected cell migration and invasion in tumors. Furthermore, the expression of linc00460 in the GSE21510 and GSE32323 was significantly elevated in patients with combined metastasis, indicating its role in promoting metastasis.

In summary, by conducting a combination analysis of methylation and RNA-seq data, we characterized the methylation-altered lncRNA in CRC, which can be used to identify the lncRNA regulatory mechanism in tumorigenesis. Integrated clinicopathological data and *in vivo* experiments confirm that the abnormally expressed LINC00460 is associated with tumor metastasis, could promote CRC cell invasion and migration, and thus may be of therapeutic values for CRC treatment.

## Data Availability

MRNA methylation and expression array data of colorectal carcinoma was acquired from published studies (GEO accession: GSE77718, GSE9348, GSE21510, GSE32323 and GSE14333).

## Author Contributions

HZ contributed to data analysis, *in vitro* experiments, paper writing, and generating tables and figures. YL contributed to the language editing and supplementary experiment. JW contributed the theoretical analysis and manuscript revision. JF contributed to final revision of the manuscript.

## Funding

This work was supported by the Jiangsu Provincial Key Research Development Program (BE2016794 to JF and BE2016795 to JW).

## Conﬂict of Interest Statement

The authors declare that the research was conducted in the absence of any commercial or financial relationships that could be construed as a potential conﬂict of interest.
