# Supplementary material for: LINC00460 Hypomethylation Promotes Metastasis in Colorectal Carcinoma
Source: Front Genet. 2019 Sep 30;10:880. doi: 10.3389/fgene.2019.00880 (PMC6779110; doi:10.3389/fgene.2019.00880)
Supplement: Supplementary file 1 [file Table_1.docx]

**Table.1. 20 lncRNAs containing both methylation and expression screened from TCGA database.**

| **LncRNAs** | **Expression** | | | **Methylation** | | |
| --- | --- | --- | --- | --- | --- | --- |
|  | **Tormal** | **Tumor** | **P-value^a^** | **Normal** | **Tumor** | **P-value^b^** |
| AC003958.2 | 0.036±0.165 | 1.562±1.818 | <0.001 | 0.591±0.117 | 0.583±0.612 | 0.564 |
| AC005256.1 | 0.131±0.415 | 2.270±1.742 | <0.001 | 0.158±0.146 | 0.163±0.536 | 0.618 |
| AC007099.1 | 0.118±0.297 | 3.950±1.970 | <0.001 | 0.345±0.029 | 0.394±0.074 | 0.003 |
| AC011754.1 | 0.177±0.656 | 0.560±1.234 | 0.158 | 0.733±0.404 | 0.540±0.154 | <0.001 |
| AFAP1-AS1 | 2.505±0.928 | 4.508±2.827 | 0.001 | 0.281±0.118 | 0.269±0.405 | 0.171 |
| CASC21 | 0.809±0.929 | 5.249±2.010 | <0.001 | 0.875±0.024 | 0.848±0.067 | 0.063 |
| ERVMER61-1 | 0.045±0.206 | 1.509±2.097 | 0.004 | 0.370±0.037 | 0.198±0.095 | <0.001 |
| FIRRE | 0.855±0.581 | 4.681±2.193 | <0.001 | 0.554±0.141 | 0.373±0.158 | <0.001 |
| HULC | 0.353±0.767 | 2.493±3.240 | 0.003 | 0.414±0.019 | 0.387±0.086 | 0.161 |
| LINC00460 | 0.376±0.687 | 4.806±1.907 | <0.001 | 0.756±0.013 | 0.615±0.099 | <0.001 |
| LINC00659 | 0.729±0.784 | 4.587±1.893 | <0.001 | 0.633±0.032 | 0.608±0.148 | 0.439 |
| LINC00858 | 0.662±0.809 | 4.832±2.388 | <0.001 | 0.226±0.008 | 0.234±0.027 | 0.203 |
| LINC01169 | 0.269±0.511 | 2.045±2.184 | <0.001 | 0.316±0.237 | 0.320±0.052 | 0.767 |
| LINC01234 | 0.788±0.768 | 4.766±3.167 | <0.001 | 0.224±0.172 | 0.188±0.471 | 0.001 |
| LINC01411 | 0.634±0.807 | 3.788±2.672 | <0.001 | 0.280±0.066 | 0.260±0.018 | <0.001 |
| LINC01511 | 0.199±0.458 | 1.951±1.828 | <0.001 | 0.350±0.069 | 0.325±0.034 | 0.001 |
| MIR205HG | 0.121±0.404 | 0.424±1.053 | 0.190 | 0.647±0.158 | 0.494±0.089 | <0.001 |
| NKX2-1-AS1 | 0.463±0.212 | 0.637±1.504 | 0.073 | 0.256±0.622 | 0.680±0.167 | 0.002 |
| NPSR1-AS1 | 0.307±0.537 | 3.549±2.252 | <0.001 | 0.358±0.113 | 0.307±0.629 | <0.001 |
| PGM5-AS1 | 4.578±1.723 | 1.027±1.149 | <0.001 | 0.260±0.414 | 0.320±0.081 | <0.001 |

^a^Difference of lncRNAs expression between tumor and normal.

^b^Difference of lncRNAs methylation between tumor and norma.
