# Supplementary material for: LINC00460 Hypomethylation Promotes Metastasis in Colorectal Carcinoma
Source: Front Genet. 2019 Sep 30;10:880. doi: 10.3389/fgene.2019.00880 (PMC6779110; doi:10.3389/fgene.2019.00880)
Supplement: Supplementary file 2 [file Table_2.docx]

**Supplementary table 2: Target genes of LINC00460 predicted by MEM database**

| **Gene name** | **Score** | **probeset id** | **Gene description** |
| --- | --- | --- | --- |
| LINC00460 | #QUERY | 1558930_AT | long intergenic non-protein coding RNA 460 [Source:HGNC Symbol;Acc:HGNC:42809] |
| LINC00460 | 3.80E-115 | 1563062_at | long intergenic non-protein coding RNA 460 [Source:HGNC Symbol;Acc:HGNC:42809] |
| LINC00973 | 7.66E-47 | 242005_at | long intergenic non-protein coding RNA 973 [Source:HGNC Symbol;Acc:HGNC:48868] |
| DCBLD2 | 6.53E-41 | 213865_at | discoidin, CUB and LCCL domain containing 2 [Source:HGNC Symbol;Acc:HGNC:24627] |
| FOSL1P1 | 1.87E-35 | 204420_at | FOS-like antigen 1 [Source:HGNC Symbol;Acc:HGNC:13718] |
| DCBLD2 | 3.33E-34 | 224911_s_at | discoidin, CUB and LCCL domain containing 2 [Source:HGNC Symbol;Acc:HGNC:24627] |
| TGFBI | 4.79E-34 | 201506_at | transforming growth factor, beta-induced, 68kDa [Source:HGNC Symbol;Acc:HGNC:11771] |
| NT5E | 2.27E-33 | 1553995_a_at | 5'-nucleotidase, ecto (CD73) [Source:HGNC Symbol;Acc:HGNC:8021] |
| GFPT2 | 2.83E-33 | 205100_at | glutamine-fructose-6-phosphate transaminase 2 [Source:HGNC Symbol;Acc:HGNC:4242] |
| NT5E | 3.71E-33 | 203939_at | 5'-nucleotidase, ecto (CD73) [Source:HGNC Symbol;Acc:HGNC:8021] |
| EFNB2 | 2.78E-31 | 202668_at | ephrin-B2 [Source:HGNC Symbol;Acc:HGNC:3227] |
| EFNB2 | 1.17E-30 | 202669_s_at | ephrin-B2 [Source:HGNC Symbol;Acc:HGNC:3227] |
| IL31RA | 3.02E-30 | 243541_at | interleukin 31 receptor A [Source:HGNC Symbol;Acc:HGNC:18969] |
| NT5E | 9.63E-30 | 1553994_at | 5'-nucleotidase, ecto (CD73) [Source:HGNC Symbol;Acc:HGNC:8021] |
| SRPX2 | 9.02E-29 | 205499_at | sushi-repeat containing protein, X-linked 2 [Source:HGNC Symbol;Acc:HGNC:30668] |
| PRKCDBP | 1.07E-28 | 213010_at | protein kinase C, delta binding protein [Source:HGNC Symbol;Acc:HGNC:9400] |
| ARNTL2 | 3.15E-28 | 224204_x_at | aryl hydrocarbon receptor nuclear translocator-like 2 [Source:HGNC Symbol;Acc:HGNC:18984] |
| ARNTL2 | 5.55E-28 | 220658_s_at | aryl hydrocarbon receptor nuclear translocator-like 2 [Source:HGNC Symbol;Acc:HGNC:18984] |
| SH3KBP1 | 7.29E-28 | 223082_at | SH3-domain kinase binding protein 1 [Source:HGNC Symbol;Acc:HGNC:13867] |
| PHLDA1 | 1.57E-27 | 225842_at | pleckstrin homology-like domain, family A, member 1 [Source:HGNC Symbol;Acc:HGNC:8933] |
| CTD-2357A8.3 | 2.38E-27 | 241394_at | N/A |
| ADORA2B | 2.91E-27 | 205891_at | adenosine A2b receptor [Source:HGNC Symbol;Acc:HGNC:264] |
| ADAM19 | 7.75E-27 | 209765_at | ADAM metallopeptidase domain 19 [Source:HGNC Symbol;Acc:HGNC:197] |
| SH3KBP1 | 5.82E-26 | 1554168_a_at | SH3-domain kinase binding protein 1 [Source:HGNC Symbol;Acc:HGNC:13867] |
| S100A2 | 6.10E-26 | 204268_at | S100 calcium binding protein A2 [Source:HGNC Symbol;Acc:HGNC:10492] |
| N/A | 7.70E-26 | 201474_s_at | N/A |
| PLAUR | 1.76E-25 | 214866_at | plasminogen activator, urokinase receptor [Source:HGNC Symbol;Acc:HGNC:9053] |
| HRH1 | 1.82E-25 | 205579_at | histamine receptor H1 [Source:HGNC Symbol;Acc:HGNC:5182] |
| SERPINE1 | 3.42E-25 | 202627_s_at | serpin peptidase inhibitor, clade E (nexin, plasminogen activator inhibitor type 1), member 1 [Source:HGNC Symbol;Acc:HGNC:8583] |
| ANXA2P2 | 8.45E-25 | 208816_x_at | annexin A2 pseudogene 2 [Source:HGNC Symbol;Acc:HGNC:539] |
| PROCR | 8.99E-25 | 203650_at | protein C receptor, endothelial [Source:HGNC Symbol;Acc:HGNC:9452] |
| KCNN4 | 9.12E-25 | 204401_at | potassium channel, calcium activated intermediate/small conductance subfamily N alpha, member 4 [Source:HGNC Symbol;Acc:HGNC:6293] |
| LETM2 | 2.14E-24 | 1552546_a_at | leucine zipper-EF-hand containing transmembrane protein 2 [Source:HGNC Symbol;Acc:HGNC:14648] |
| PHLDA1 | 2.17E-24 | 217998_at | pleckstrin homology-like domain, family A, member 1 [Source:HGNC Symbol;Acc:HGNC:8933] |
| AMIGO2 | 2.33E-24 | 222108_at | adhesion molecule with Ig-like domain 2 [Source:HGNC Symbol;Acc:HGNC:24073] |
| HMGA2 | 2.76E-24 | 208025_s_at | high mobility group AT-hook 2 [Source:HGNC Symbol;Acc:HGNC:5009] |
| PLAUR | 2.80E-24 | 210845_s_at | plasminogen activator, urokinase receptor [Source:HGNC Symbol;Acc:HGNC:9053] |
| GLIPR1 | 3.45E-24 | 204222_s_at | GLI pathogenesis-related 1 [Source:HGNC Symbol;Acc:HGNC:17001] |
| TIMP1 | 3.52E-24 | 201666_at | TIMP metallopeptidase inhibitor 1 [Source:HGNC Symbol;Acc:HGNC:11820] |
| MET | 4.50E-24 | 213816_s_at | MET proto-oncogene, receptor tyrosine kinase [Source:HGNC Symbol;Acc:HGNC:7029] |
| LOXL2 | 5.27E-24 | 202998_s_at | lysyl oxidase-like 2 [Source:HGNC Symbol;Acc:HGNC:6666] |
| ST3GAL6 | 6.75E-24 | 230175_s_at | ST3 beta-galactoside alpha-2,3-sialyltransferase 6 [Source:HGNC Symbol;Acc:HGNC:18080] |
| PLAUR | 7.90E-24 | 211924_s_at | plasminogen activator, urokinase receptor [Source:HGNC Symbol;Acc:HGNC:9053] |
| PHLDA1 | 8.89E-24 | 217999_s_at | pleckstrin homology-like domain, family A, member 1 [Source:HGNC Symbol;Acc:HGNC:8933] |
| EMP3 | 1.02E-23 | 203729_at | epithelial membrane protein 3 [Source:HGNC Symbol;Acc:HGNC:3335] |
| GLIPR1 | 1.06E-23 | 226136_at | GLI pathogenesis-related 1 [Source:HGNC Symbol;Acc:HGNC:17001] |
| STEAP1 | 2.37E-23 | 205542_at | six transmembrane epithelial antigen of the prostate 1 [Source:HGNC Symbol;Acc:HGNC:11378] |
| EREG | 2.73E-23 | 205767_at | epiregulin [Source:HGNC Symbol;Acc:HGNC:3443] |
| ANXA2 | 3.34E-23 | 213503_x_at | annexin A2 [Source:HGNC Symbol;Acc:HGNC:537] |
| CATSPER1 | 4.30E-23 | 1552335_at | cation channel, sperm associated 1 [Source:HGNC Symbol;Acc:HGNC:17116] |
| PHLDA1 | 7.07E-23 | 217996_at | pleckstrin homology-like domain, family A, member 1 [Source:HGNC Symbol;Acc:HGNC:8933] |
| MET | 1.28E-22 | 203510_at | MET proto-oncogene, receptor tyrosine kinase [Source:HGNC Symbol;Acc:HGNC:7029] |
| PRKCA | 1.64E-22 | 215195_at | protein kinase C, alpha [Source:HGNC Symbol;Acc:HGNC:9393] |
| ELK3 | 1.97E-22 | 221773_at | ELK3, ETS-domain protein (SRF accessory protein 2) [Source:HGNC Symbol;Acc:HGNC:3325] |
| ITGA5 | 2.05E-22 | 201389_at | integrin, alpha 5 (fibronectin receptor, alpha polypeptide) [Source:HGNC Symbol;Acc:HGNC:6141] |
| CAV1 | 2.37E-22 | 203065_s_at | caveolin 1, caveolae protein, 22kDa [Source:HGNC Symbol;Acc:HGNC:1527] |
| S100A16 | 3.46E-22 | 227998_at | S100 calcium binding protein A16 [Source:HGNC Symbol;Acc:HGNC:20441] |
| LGALS1 | 3.60E-22 | 201105_at | lectin, galactoside-binding, soluble, 1 [Source:HGNC Symbol;Acc:HGNC:6561] |
| HPCAL1 | 4.11E-22 | 212552_at | hippocalcin-like 1 [Source:HGNC Symbol;Acc:HGNC:5145] |
| N/A | 7.98E-22 | 227486_at | N/A |
| AHNAK2 | 8.25E-22 | 212992_at | AHNAK nucleoprotein 2 [Source:HGNC Symbol;Acc:HGNC:20125] |
| PMEPA1 | 1.13E-21 | 222449_at | prostate transmembrane protein, androgen induced 1 [Source:HGNC Symbol;Acc:HGNC:14107] |
| RP11-30P6.6 | 1.25E-21 | 234219_at | N/A |
| SHC1 | 1.32E-21 | 214853_s_at | SHC (Src homology 2 domain containing) transforming protein 1 [Source:HGNC Symbol;Acc:HGNC:10840] |
| SLC20A1 | 1.46E-21 | 201920_at | solute carrier family 20 (phosphate transporter), member 1 [Source:HGNC Symbol;Acc:HGNC:10946] |
| HKDC1 | 2.03E-21 | 227614_at | hexokinase domain containing 1 [Source:HGNC Symbol;Acc:HGNC:23302] |
| CACNA2D4 | 2.39E-21 | 228083_at | calcium channel, voltage-dependent, alpha 2/delta subunit 4 [Source:HGNC Symbol;Acc:HGNC:20202] |
| STARD3NL | 2.41E-21 | 223065_s_at | STARD3 N-terminal like [Source:HGNC Symbol;Acc:HGNC:19169] |
| ANXA2 | 2.68E-21 | 201590_x_at | annexin A2 [Source:HGNC Symbol;Acc:HGNC:537] |
| ARL4C | 3.62E-21 | 202208_s_at | ADP-ribosylation factor-like 4C [Source:HGNC Symbol;Acc:HGNC:698] |
| GLIPR1 | 4.74E-21 | 226142_at | GLI pathogenesis-related 1 [Source:HGNC Symbol;Acc:HGNC:17001] |
| FAM126A | 5.07E-21 | 223625_at | family with sequence similarity 126, member A [Source:HGNC Symbol;Acc:HGNC:24587] |
| ANXA2 | 6.40E-21 | 210427_x_at | annexin A2 [Source:HGNC Symbol;Acc:HGNC:537] |
| FRMD5 | 7.53E-21 | 230831_at | FERM domain containing 5 [Source:HGNC Symbol;Acc:HGNC:28214] |
| HMGA2 | 8.02E-21 | 1558683_a_at | high mobility group AT-hook 2 [Source:HGNC Symbol;Acc:HGNC:5009] |
| TNFRSF12A | 8.91E-21 | 218368_s_at | tumor necrosis factor receptor superfamily, member 12A [Source:HGNC Symbol;Acc:HGNC:18152] |
| ECM1 | 9.18E-21 | 209365_s_at | extracellular matrix protein 1 [Source:HGNC Symbol;Acc:HGNC:3153] |
| PLEK2 | 9.98E-21 | 218644_at | pleckstrin 2 [Source:HGNC Symbol;Acc:HGNC:19238] |
| PMEPA1 | 1.04E-20 | 217875_s_at | prostate transmembrane protein, androgen induced 1 [Source:HGNC Symbol;Acc:HGNC:14107] |
| S100A3 | 1.04E-20 | 206027_at | S100 calcium binding protein A3 [Source:HGNC Symbol;Acc:HGNC:10493] |
| TMSB10 | 1.09E-20 | 217733_s_at | thymosin beta 10 [Source:HGNC Symbol;Acc:HGNC:11879] |
| LAMB3 | 1.32E-20 | 209270_at | laminin, beta 3 [Source:HGNC Symbol;Acc:HGNC:6490] |
| CACNA2D4 | 1.38E-20 | 1552690_a_at | calcium channel, voltage-dependent, alpha 2/delta subunit 4 [Source:HGNC Symbol;Acc:HGNC:20202] |
| PXN | 1.45E-20 | 201087_at | paxillin [Source:HGNC Symbol;Acc:HGNC:9718] |
| TMEM158 | 1.69E-20 | 213338_at | transmembrane protein 158 (gene/pseudogene) [Source:HGNC Symbol;Acc:HGNC:30293] |
| ARNTL2 | 1.70E-20 | 223586_at | aryl hydrocarbon receptor nuclear translocator-like 2 [Source:HGNC Symbol;Acc:HGNC:18984] |
| LAMC2 | 1.71E-20 | 202267_at | laminin, gamma 2 [Source:HGNC Symbol;Acc:HGNC:6493] |
| OSBPL3 | 1.88E-20 | 209626_s_at | oxysterol binding protein-like 3 [Source:HGNC Symbol;Acc:HGNC:16370] |
| TGFA | 2.28E-20 | 205016_at | transforming growth factor, alpha [Source:HGNC Symbol;Acc:HGNC:11765] |
| DPY19L1 | 2.32E-20 | 212792_at | dpy-19-like 1 (C. elegans) [Source:HGNC Symbol;Acc:HGNC:22205] |
| STX1A | 2.78E-20 | 204729_s_at | syntaxin 1A (brain) [Source:HGNC Symbol;Acc:HGNC:11433] |
| IRAK2 | 4.95E-20 | 231779_at | interleukin-1 receptor-associated kinase 2 [Source:HGNC Symbol;Acc:HGNC:6113] |
| S100A11 | 5.80E-20 | 200660_at | S100 calcium binding protein A11 [Source:HGNC Symbol;Acc:HGNC:10488] |
| SH3KBP1 | 6.08E-20 | 235692_at | SH3-domain kinase binding protein 1 [Source:HGNC Symbol;Acc:HGNC:13867] |
| FAM129B | 6.67E-20 | 223019_at | family with sequence similarity 129, member B [Source:HGNC Symbol;Acc:HGNC:25282] |
| NME7 | 7.03E-20 | 219553_at | NME/NM23 family member 7 [Source:HGNC Symbol;Acc:HGNC:20461] |
| S100A6 | 7.16E-20 | 217728_at | S100 calcium binding protein A6 [Source:HGNC Symbol;Acc:HGNC:10496] |
| ARL4C | 8.43E-20 | 202207_at | ADP-ribosylation factor-like 4C [Source:HGNC Symbol;Acc:HGNC:698] |
| PLP2 | 1.26E-19 | 201136_at | proteolipid protein 2 (colonic epithelium-enriched) [Source:HGNC Symbol;Acc:HGNC:9087] |
| SYNJ2 | 1.30E-19 | 212828_at | synaptojanin 2 [Source:HGNC Symbol;Acc:HGNC:11504] |
| STK17A | 1.46E-19 | 202693_s_at | serine/threonine kinase 17a [Source:HGNC Symbol;Acc:HGNC:11395] |
